# Leukemia Cutis As the Initial Manifestation of Chronic Lymphocytic Leukemia Progression

**DOI:** 10.7759/cureus.33013

**Published:** 2022-12-27

**Authors:** Wilfredo M Pedreira-García, Dalianie Nieves, Marlian Montesinos-Cartagena, Carlos A Cortés, William Cáceres-Perkins, José Rabelo-Cartagena

**Affiliations:** 1 Internal Medicine, VA (Veterans Affairs) Caribbean Healthcare System, San Juan, PRI; 2 Hematology and Oncology, San Juan City Hospital, San Juan, PRI; 3 Nephrology, VA (Veterans Affairs) Caribbean Healthcare System, San Juan, PRI; 4 Hematology and Oncology, VA (Veterans Affairs) Caribbean Healthcare System, San Juan, PRI; 5 Dermatology, VA (Veterans Affairs) Caribbean Healthcare System, San Juan, PRI

**Keywords:** non-hodgkin’s lymphoma b-cell, rash cutaneous lesions, atypical rash, chronic lymphocytic leukemia (cll), leukemia cutis

## Abstract

Chronic lymphocytic leukemia (CLL) is a malignant proliferation of monoclonal mature B-cells in peripheral blood. Leukemia cells can commonly spread from the blood to other sites such as the lymph nodes, liver, and spleen. However, contrary to T-cell lymphomas that can involve the skin, CLL metastasis to the skin is unusual and is rarely the first manifestation of systemic disease. When leukemia cells invade the skin, it is termed leukemia cutis. Furthermore, multiple skin morphologies can be present in leukemia cutis making diagnosis challenging. Likewise, it can be mistaken for other common etiologies such as drug or substance allergy, infection, and scabies, among others. We herein present a case of CLL with leukemia cutis as the initial manifestation of systemic disease. The initial punch biopsy results were non-specific for inflammatory changes, but a subsequent biopsy revealed findings confirming leukemia cutis. This case not only demonstrates that identifying malignant skin manifestations in a timely manner and treating them is essential, as it improves the quality of life and survival, but also demonstrates that leukemia cutis can be a dynamic disease where multiple biopsies may be needed to confirm the diagnosis, as histopathology can change over time.

## Introduction

Chronic lymphocytic leukemia is part of the subset of non-Hodgkin lymphomas that is characterized by the accumulation of mature monoclonal B-cells in blood [1]. It is usually diagnosed when there is an increase in absolute lymphocyte counts above 5x10^9^/L in peripheral blood with flow cytometry confirming monoclonality of B-cells. From flow cytometry, further cell surface markers can be obtained to aid in diagnosis. These include Kappa/Lambda, CD5, CD10, CD19, CD20, and CD23 markers or fluorescence in situ hybridization (FISH) for t(11;14);t(11q;v) [2]. Leukemia cells initially form in the bone marrow and then enter the bloodstream. In chronic lymphocytic leukemia (CLL), this process is usually indolent, and most patients are asymptomatic at diagnosis. On the other hand, 10% of patients can present with B symptoms (fever, night sweats, weight loss, among others) while 20% to 50% of the patients can exhibit hepatosplenomegaly [3].

However, contrary to T-cell lymphomas that can involve the skin (as seen in mycosis fungoides or Sezary syndrome), CLL metastasis to the skin is unusual and is rarely the first manifestation of systemic disease [4]. When cutaneous neoplastic infiltration of leukemia occurs, it is termed leukemia cutis (LC). The exact incidence of LC in CLL is unknown, but it is seen in less than 5% of cases [1]. On a systematic search from 2000 to 2019 by Aldapt and Yassin, only 27 of 56 cases (55.1%) of LC by CLL reported in the literature had primary cutaneous involvement [3]. Likewise, skin morphology can manifest in numerous ways, including solitary, grouped, or diffuse papules, plaques, nodules, or large tumors. More rarely, it may include erythroderma, palmar plaques, or chronic paronychia [4]. In view of the different morphologies and the rarity of the disease, skin eruptions may be mistaken for other common causes. We herein present a case of leukemia cutis by CLL presenting initially as multiple erythematous papules (some forming linear patterns) on upper extremities and erythematous patches on legs of different sizes that were initially mistaken as dermatitis and scabies, which delayed correct treatment.

## Case presentation

**Figure 1:**
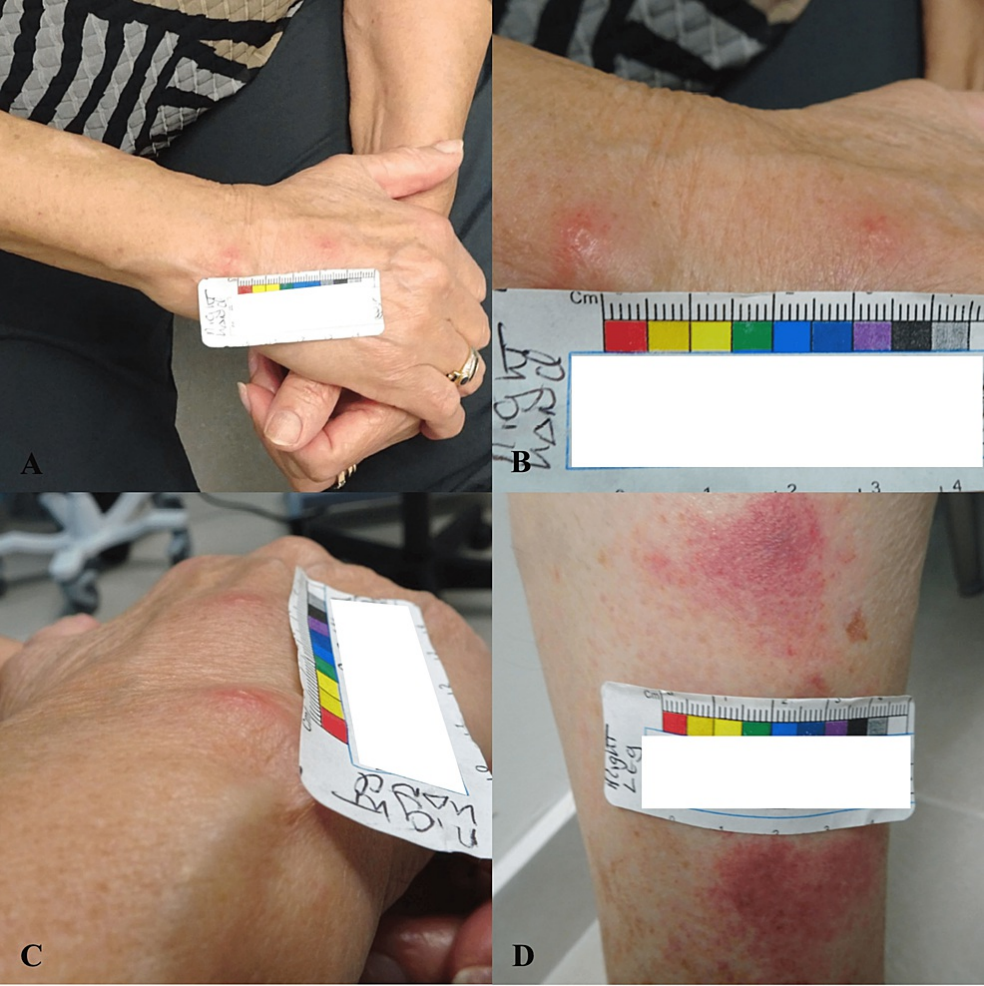
Multiple skin papules’ presentation at six months (A) Right upper extremity with two papules. (B) Close-up of the right-hand papules. (C) Papules on the right hand from another perspective. (D) Right lower extremities with multiple patches.

This is the case of a 78-year-old Hispanic woman with a medical history of CLL diagnosed one year ago. The baseline WBC count was 20-30x10^3^/µL (Table 1) with initial flow cytometry revealing high positivity of ZAP-70 and FISH positive for p53 deletion, indicative of a poor prognosis. The patient was being followed by the Hematology/Oncology department with frequent primary care physician follow-ups. Since the initial diagnosis, laboratory results did not elucidate anemia or thrombocytopenia. Physical examination and imaging findings were negative for visceromegaly. Baseline positron emission tomography/computed tomography (PET/CT) from one year prior did not show 18F-fluorodeoxyglucose (FDG)-avid lesions and demonstrated borderline-size lymph nodes without a significant abnormal increase in metabolic activity. In view of no systemic manifestations, and stable WBC count (20K-30K) the patient met the criteria for Rai Stage 0 CLL, and surveillance was recommended. Nevertheless, six months later, she presented with pruritic, red papules and patches around the dorsum and palmar aspects of her hands, feet, and neck (Figure 1). Hydrocortisone, diphenhydramine, and a change in detergents were recommended by the primary care physician, but symptoms persisted. Scabies was also in the differential diagnoses due to small linear burrow-like lesions seen in the palmar and dorsal aspects of her hands, thus permethrin was also prescribed without improvement of symptoms.

**Table 1:** Comparison of CBC with differential at the initial hematology/oncology visit, six months, and 12 months

CBC	Initial Visit	6 months later	12 months later	Units	Reference Ranges
White Blood Cells	23.1	35.6	68.0	x10^3^/µl	4.3-9.3
Red Blood Cells	5.0	4.8	4.0	x10^6^/µl	4.7-6.1
Hemoglobin	12.6	12.4	10.0	g/dL	12.6-17.8
Hematocrit	40.6	40.2	32.4	%	37.9-54.5
Mean Corpuscular Volume	81.0	83.1	80.6	fL	81-102
Mean Corpuscular Hemoglobin	25.1	25.6	24.9	pg	26-34
Mean Corpuscular Hemoglobin Concentration	31.0	30.8	30.9	g/dL	31-36.5
Red Cell Distribution Width	16.2	16.3	17.6	%	11-15
Platelet	180	145.0	203	x10^3^/µl	155-371
Mean Platelet Volume	11.6	12.0	11.5	fL	9-12.9
Bands	1.0	0.0	0.0	%	0-9
Segmented	15.0	18.0	5.0	%	34-74
Lymphocytes	70.0	68.0	93.0	%	17-48
Monocytes	5.0	13.0	2.0	%	2-12
Eosinophils	5.0	1.0	0.0	%	0-1.4

**Figure 2:**
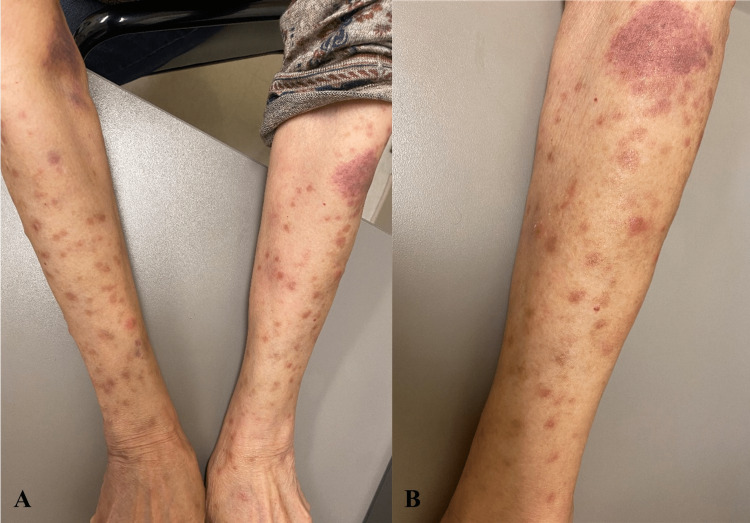
Skin leukemia cutis presentation at 12 months (A) Bilateral arms with multiple pink and brown papules and macules with a left-sided patch. (B) Magnified left arm.

**Figure 3:**
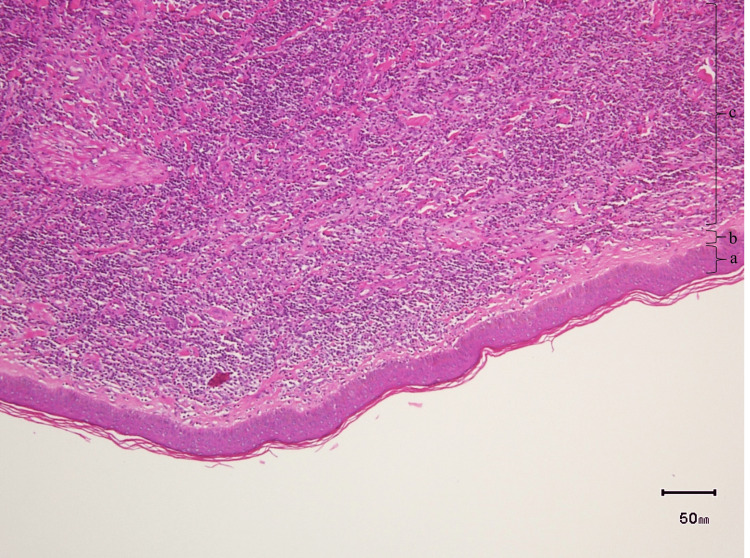
Histology of punch skin biopsy H&E magnified 10x: (a) Uninvolved epidermis; (b) Narrow area of the papillary dermis uninvolved by an infiltrate (Grenz zone); (c) Diffuse dense dermal lymphohistiocytic infiltrate.

In view of the persistence of lesions and symptoms, the patient was referred to a dermatologist where an initial skin biopsy was taken from a left ventral forearm papule. The dermatopathologist report concluded that the sample was positive for a mixed-cell inflammatory infiltrate with eosinophils, indicative of a non-specific inflammatory process. On a follow-up outpatient hematology/oncology visit, the patient’s rash progressed from multiple small papules and patches to pink and brown macules and papules (Figure 2). The patient had an increased doubling time of her leukocytosis and started to develop anemia, changing staging to Rai Stage III. In view of the high disease burden, the patient was started on anti-CD 20 therapy with obinutuzumab-acalabrutinib. After the initial cycle, her skin rash improved with decreased erythema and pruritus. On a follow-up visit with another dermatologist, a punch skin biopsy was repeated. Results were positive for dense dermal lymphohistiocytic infiltrate separated by a Grenz zone from the epidermis (Figure 3) with B-cells positive for CD5, CD23, CD-43, and BCL-2, and scattered T-cells. These findings are consistent with the cutaneous involvement of CLL, and LC was confirmed.

## Discussion

CLL progression can manifest as progressive bone marrow failure (anemia, thrombocytopenia), progressive or symptomatic splenomegaly, lymphadenopathy, progressive lymphocytosis with an increase of >50% over a two-month period or lymphocyte doubling time in less than six months, fatigue, night sweats, and unintentional weight loss, among others [5]. The cutaneous manifestations of CLL may be classified as specific if they are caused by direct seeding of B-cells or as nonspecific if they are secondary to the increased risk posed by CLL to develop skin lesions. For instance, Leukemia Cutis and Richter’s syndrome are specific CLL cutaneous manifestations while Sweet’s syndrome, urticaria, and exfoliative erythroderma are nonspecific manifestations [6].

When LC is present, clinical manifestation varies and may present as papules, nodules, or tumors. To a lesser degree, other manifestations can include erythroderma, chronic paronychia, palmar plaques, vesiculobullous eruptions, and subungual lesions [4]. It has also been described as papulonodular skin lesions, erythematous patches, and ulceration in a minority of cases. The most common skin site manifestations include the head and neck (33.9% of lesions), followed by the trunk and extremities (26.8%) [3].

Disease onset of LC generally appears months to years following CLL diagnosis with an average delay of 39 months between diagnosis and presentation [3-4]. In the case of our patient, she started to develop multiple erythematous papules on her trunk and extremities six months after diagnosis of CLL, which rapidly progressed to pink and brown macules. Concomitantly, she had an increase in the doubling time of lymphocyte count and anemia. In the first skin punch biopsy, findings were non-specific while the latter was positive for dense dermal lymphohistiocytic infiltrate confirming the presence of B-cells. Histological confirmation of LC may be challenging. In a study done by Thiesen et al., they were only able to confirm LC histologically in 3% of their CLL cases with skin manifestations [7]. Furthermore, three main architectural patterns of CLL present on histopathology have been described by Cerroni et al. [4,8]. The first is the patchy perivascular and periadnexal pattern where there is a dense infiltrate of lymphoid cells around the vessels and adnexal structures within the dermis and subcutaneous fat. The second pattern is the nodular diffuse where one or more nodules of neoplastic cells in the dermis or subcutaneous tissue are seen. Lastly, and considered the rarest, the band-like pattern is described as a dense infiltrate of lymphoid cells that arrange in a band form in the superficial and middle dermis. As per Cerroni et al., the same patient may manifest different histological patterns through different points in time [4,9]. For this reason, LC can be considered a dynamic condition and multiple biopsies may be needed to confirm the diagnosis, as seen in this patient where dense dermal lymphohistiocytic infiltrates with perivascular and periadnexal pattern was observed.

Rai Clinical Staging is used to predict CLL progression and helps clinicians determine when to initiate treatment. Stratification is based on whether an individual has or does not have any of the following characteristics: lymphadenopathy, hepatomegaly, splenomegaly, anemia, or thrombocytopenia. It is classified from Rai 0 (patient with lymphocytosis but without of the any above-mentioned characteristics; >10-year overall survival) to Rai IV (having thrombocytopenia of ≤100,000/mm^3^; <4 years overall survival) (Table 2) [2,9]. Our patient had a CLL Rai Stage 0 both during initial diagnosis and rash onset, which is why observation was recommended [2,5]. Later, she developed worsening skin eruptions with subsequent anemia, progressing from Rai Stage 0 to Rai Stage III. Thus, treatment was initiated with obinutuzumab-acalabrutinib as per guidelines [2,5] with an improvement in skin eruptions. This is consistent with the literature review where most patients show complete resolution of skin lesions after treatment. Hence, as seen in our patient, most patients attain complete or partial cutaneous remission with chemotherapy [3]. Interestingly, our patient presented with rash months prior to disease progression. For this reason, in patients where the rash is the primary manifestation of CLL, treatment initiation may be considered despite low Rai Staging.

**Table 2:** Rai Clinical Staging for CLL progression CLL: chronic lymphocytic leukemia

Clinical stage	Clinical findings	Risk	Overall survival
Rai Stage 0	Lymphocytosis	Low	> 10 years
Rai Stage I	Lymphocytosis with enlarged lymph nodes	Intermediate	7 years
Rai Stage II	Lymphocytosis with splenomegaly or hepatomegaly	Intermediate	7 years
Rai Stage III	Lymphocytosis with anemia (≤11 g/L)	High	<4 years
Rai Stage IV	Lymphocytosis with thrombocytopenia (≤100,000/mm^3^)	High	<4 years

## Conclusions

CLL with cutaneous manifestations is rare and difficult to diagnose when it is the initial presentation. When a rash presents, the most common etiologies and their respective treatments are usually sought out first. Nevertheless, if the rash persists in CLL despite no change in Rai Staging, skin malignancy should be in the differential diagnosis and a skin biopsy is highly recommended with follow-up of routine laboratories and physical examinations. In this case, despite an initial inconclusive biopsy, a rash was present six months after the initial diagnosis, and then six months later, laboratory tests demonstrated worsening disease, requiring anti-CD20 therapy. A repeated biopsy was able to confirm the LC diagnosis. In this manner, when malignant skin manifestations are suspected and biopsy of lesions is initially non-specific (as rashes can be dynamic) despite Rai Staging, treatment should not be delayed.

## References

[1] Raufi A, Alsharedi M, Khelfa Y, Griswold DC, Lebowicz Y (2016). Leukemia cutis in a patient with chronic lymphocytic leukemia presenting as bilateral helical nodules. SAGE Open Med Case Rep.

[2] Wierda WG, Brown J, Abramson JS (2022). NCCN Guidelines® insights: chronic lymphocytic leukemia/small lymphocytic lymphoma, version 3.2022. J Natl Compr Canc Netw.

[3] Aldapt MB, Yassin M (2021). Leukemia cutis as an early presentation or relapsing manifestation of chronic lymphocytic leukemia (CLL). Acta Biomed.

[4] Robak E, Robak T (2007). Skin lesions in chronic lymphocytic leukemia. Leuk Lymphoma.

[5] Hallek M, Al-Sawaf O (2021). Chronic lymphocytic leukemia: 2022 update on diagnostic and therapeutic procedures. Am J Hematol.

[6] Fried LJ, Criscito MC, Stevenson ML, Pomeranz MK (2022). Chronic lymphocytic leukemia and the skin: implications for the dermatologist. Int J Dermatol.

[7] Thiesen I, Wehkamp U, Brüggemann M, Ritgen M, Murga Penas EM, Klapper W, Oschlies I (2019). Skin involvement by chronic lymphocytic leukaemia is frequently associated with unrelated neoplastic or inflammatory cutaneous disease and is not indicative of general disease progression. Br J Dermatol.

[8] Cerroni L, Zenahlik P, Höfler G, Kaddu S, Smolle J, Kerl H (1996). Specific cutaneous infiltrates of B-cell chronic lymphocytic leukemia. A clinicopathologic and prognostic study of 42 patients. Am J Surg Pathol.

[9] Rai KR, Sawitsky A, Cronkite EP (1975). Clinical staging of chronic lymphocytic leukemia. Blood.

